# Family physician perceptions of working with LGBTQ patients: physician training needs

**Published:** 2015-04-20

**Authors:** Brenda Beagan, Erin Fredericks, Mary Bryson

**Affiliations:** 1 School of Occupational Therapy, Dalhousie University, Halifax, NS; 2 Sociology Department, St. Thomas University, Fredericton, NB; 3 Institute for Gender, Race, Sexuality, and Social Justice, University of British Columbia, Vancouver, BC

## Abstract

**Background:**

Medical students and physicians report feeling under-prepared for working with patients who identify as lesbian, gay, bisexual, transgender or queer (LGBTQ). Understanding physician perceptions of this area of practice may aid in developing improved education.

**Method:**

In-depth interviews with 24 general practice physicians in Halifax and Vancouver, Canada, were used to explore whether, when and how the gender identity and sexual orientation of LGBTQ women were relevant to good care. Inductive thematic analysis was conducted using ATLAS.ti data analysis software.

**Results:**

Three major themes emerged: 1) Some physicians perceived that sexual/gender identity makes little or no difference; treating every patient as an individual while avoiding labels optimises care for everyone. 2) Some physicians perceived sexual/gender identity matters primarily for the provision of holistic care, and in order to address the effects of discrimination. 3) Some physicians perceived that sexual/gender identity both matters and does not matter, as they strove to balance the implications of social group membership with recognition of individual differences.

**Conclusions:**

Physicians may be ignoring important aspects of social group memberships that affect health and health care. The authors hold that individual and socio-cultural differences are both important to the provision of quality health care. Distinct from stereotypes, generalisations about social group differences can provide valuable starting points, raising useful lines of inquiry. Emphasizing this distinction in medical education may help change physician approaches to the care of LGBTQ women.

## Introduction

As a relatively invisible patient population, lesbian, gay, bisexual, transgendered and queer (LGBTQ) people have unique healthcare needs and associated risks that remain under-acknowledged.^1^–^8^ Physician uncertainty and discomfort regarding working with LGBTQ patients, as well as lack of LGBTQ-specific health knowledge, compromise care: many patients experience clinical encounters as heterosexist, inhospitable and stigmatizing;^9^–^11^ standards for best practice are rudimentary;^6^ and physicians frequently question the relevance of sexual or gender identity to health care.^12^ Not surprisingly, in Canada LGBTQ persons are twice as likely as other individuals to *not* have a family doctor and are significantly less likely to seek out health care.^13^,^14^ When they do seek medical attention, they frequently experience difficulty revealing their sexual or gender identity, further compromising care.^15^–^17^ Part of the reason for not seeking care, and not disclosing sexual or gender identity, are negative previous health care experiences and anticipated responses from physicians.^6^

Physicians have traditionally received little education concerning LGBTQ health.^13^,^18^,^19^ Medical students cite lack of training as a reason they are particularly uncomfortable asking about a patient’s sexual orientation or gender identity,^20^ feeling forced to fall back upon personal experience.^21^ A recent survey of North American medical schools found that most curricula teach students to ask if patients have sex with men, women or both, but rarely go beyond this or address issues of identity.^22^ The hidden curriculum may unintentionally undermine training; in a recent cross-Canada survey students reported that teachers and role models routinely expressed negative biases against homosexuals.^23^

Given that medical education in this area is still inadequate, and given the importance of role models in medical education,^24^ an understanding of physician perceptions of LGBTQ care is important to aid in developing improved education in this area. Based on in-depth interviews, this paper sought to interpretively analyze the experiences and understandings of general practice physicians in two Canadian cities (Halifax and Vancouver) about their work with women patients who identify as LGBTQ. Focusing on participants’ rationales for how they worked with LGBTQ patients, it particularly examines when gender identity and sexual orientation were deemed to matter, and how.

## Methods

### Study design, methodology, sampling

This qualitative study drew from both critical phenomenology^25^,^26^ and ethnographic traditions of thick description,^27^ interviewing 24 family physicians using semi-structured face-to-face interviews of 60–90 minutes each. The intent was not theory generation, but rather interpretive analysis of participant experience, perceptions and narratives. Following research ethics approval from University of British Columbia and Dalhousie University, recruitment was conducted through advertisements in local clinics, letters sent through physician mailing lists, posters and ads in LGBTQ venues, word of mouth and snowball sampling. Recruitment simply sought participants with experience working with LGBTQ patients. This was part of a larger study that included interviews with patients and with nurses; to increase homogeneity of the sample for greater thematic saturation, only LGBTQ patients who identified as women were recruited in that portion of the study. Thus interviews with physicians analysed for this paper focused on their work with LGBTQ *women* patients.

### Data collection

After discussing informed consent, physicians were interviewed one-on-one asking how they experienced and understood primary health care practice with LGBTQ women. The semi-structured interview guide asked about physicians’ experiences working with LGBTQ women patients, when they felt most comfort and discomfort, what training they had had and in what areas they might like more training. Each participant was assigned a pseudonym.

### Data analysis and rigor

Interviews were audio recorded, transcribed verbatim, and analyzed inductively, generating themes and sub-themes which were coded using ATLAS.ti qualitative data analysis software. The software is valuable for multiple qualitative methodologies, providing a tool for data management and systematic approaches to analysis.

Each transcript was read repeatedly by members of the team, discussing the narratives it contained and creating memos to distill each participant’s story. Having examined several transcripts the team collectively generated themes and sub-themes to code the data.^28^ Interview segments were interpreted both in the context of the larger interview, and in comparison with other transcripts. Coding was conducted by the team of researchers and research assistants, seeking consensus on codes and interpretations, and discussing individual transcripts at weekly meetings. Summaries of preliminary analyses were returned to participants for feedback. Drawing on the coded data, and again returning to transcripts, the analyses in this paper particularly drew on codes concerning ‘difference,’ ‘when difference matters’ and ‘assumptions,’ subjecting all three to further interpretive analysis by the lead author.

### Sample

Of the 24 physician participants, most identified as heterosexual women, with five heterosexual men and one gay man. Two of the women identified as LGBTQ. No one identified as transgender. The family physicians worked in clinics and private practice, had practiced 7–40 years, and all self-identified as working to some extent with LGBTQ patients.

## Results

Though we asked these physicians about their work with LGBTQ women, they often responded by discussing LGBTQ care more broadly. Therefore we report what our participants said about LGBTQ patient care generally even though we asked specifically about LGBTQ women. Nineteen of the 24 physicians said they had learned little or nothing about LGBTQ health (especially transgender health) at medical school. One had only learned about homosexuality as a psychiatric diagnosis, another had only heard about lesbians in derogatory comments from physicians during clinical rotations. Positive learning had come from patients and colleagues, occasionally continuing professional development such as conferences, and reading. Some said they had learned effective communication skills and a patient-centered approach which should be effective with any patient. Some argued that there is better teaching in schools now than when they were students, though a few noted that social and cultural issues are still not taken seriously:

The humanity part of it is so important. …And it’s undervalued. The [social awareness] course that we had everybody hated, and everybody still hates, I still hear about it. I mean, partly it’s not done very well, but partly people don’t take it seriously. They don’t think it’s important. (Jacqueline, Vancouver)

Five participants had learned about LGBTQ health because they deliberately selected electives or residencies where they encountered LGBTQ patients or strong mentors.

Three major themes emerged concerning perceptions of sexual/gender identity in health care. First, *it makes no difference* (sub-themes: except for specific health issues; everyone is an individual; avoid labels). Second, *sexual/gender identity matters* (sub-themes: for holistic care; because of discrimination). Third, *it matters, but it doesn’t matter*. Each of these themes is discussed below.

### It really makes no difference

Most commonly, participants suggested there are no significant differences between primary care for LGBTQ women and care for any other patients. In other words, sexual orientation and gender identity were seen as largely irrelevant to care provision, because physicians treat everyone equally, treat everyone the same. For example Nancy (Vancouver) said, “I understand it’s important to that patient. But to me, I guess it doesn’t impact the way I practice, because I wouldn’t do anything different. I’d feel that I would be treating everybody equally.” Liza (Halifax) echoed this point: “I’m doing many of the same things with everybody regardless of orientation or gender.” Richard (Halifax) went so far as to say, “I’m not sure I need to know ... I don’t see anything that changes.”

Some suggested the only way it matters concerns health issues specific to sexual practices or gender transitioning. For example, physical examination, blood test results, cardiac monitoring would all be affected if a transgender patient were on hormone therapy. Some physicians thought it mattered in that some routine exams may not be needed for LGBTQ patients; a surprising number of physicians questioned whether women who have never had sex with men need pap smears.

#### Identity doesn’t matter, only sexual practices matter

Several participants saw sexual health as the primary area where sexual orientation might have an effect. This led some to focus less on sexual/gender identity and more on sexual practices. One physician argued it does not matter whether or not she knows a patient identifies as LGBTQ, because she asks everyone about sexual practices: “If I’m talking about HPV, I’ll say ‘Look, there’s oral sex, genital sex, anal sex, and digital sex. And depending on what you do, this is how the virus is transmitted, so here’s your risk’” (Helen, Halifax). In general, participants tended to focus on sexual health when they identified any aspects of care that might differ for LGBTQ patients, including discussion of STIs, HPV, cervical cancer and pap smears, birth control and pregnancy. For example, birth control might not be relevant to women who only have sex with women, but pregnancy is likely to be somewhat complicated. Occasionally mental health was raised as relevant to LGBTQ patients, noting that mental health issues may be more prevalent in that population.

#### Everyone is an individual

One of the main reasons physicians offered for why sexual and gender identity do not matter was because they treat every patient as a unique individual. For example, one physician said:

Every patient is different irrespective of their sexual orientation or their race or anything. Each person that comes in is a unique patient; it doesn’t mean you can’t treat them holistically and objectively. ... You just have to use the principles that you’ve been taught and adapt it to each patient differently. (Sarah, Halifax)

Some participants mentioned accomplishing this task of seeing everyone as unique individuals by never making assumptions about anyone. They asked questions about circumstances and options available, rather than about social identities, so as not to assume anything. Some emphasised that they always strive to suspend any prejudices, remaining non-judgmental with all patients, and not getting “distracted” by things about a patient that the physicians personally find challenging.

I am not very comfortable with any decisions made to change one’s body using hormones and surgical treatments. I try not to let my prejudices get in the way. ... I don’t see that half hearted mutilation is of any benefit. I don’t want to share these feelings as I am not in the field and don’t appreciate the benefits attained by these patients. ... I will be non-judgmental treating them. But at the same time wondering why. (John, Vancouver)

The assumption here is that if (negative) judgments are well-hidden they have no impact on patients, and are therefore unproblematic. The description of a transgender patient’s medical and surgical transition processes as ‘distractions’ from what really matters in the health care encounter suggests the focus on individualism negates the importance of social identities.

#### Avoid labels

Participants particularly avoided labels, suggesting that labeling social identities is akin to making assumptions about individual patients, using stereotypes and prejudging people. Some avoided labels by focusing on practices rather than identities, in order to avoid making any assumptions:

That’s one thing that is important to me, is not to label people. They’re people. People have choices and of those– So that’s how I would approach it, is ‘In your choice, do you prefer a same sex partner or do you prefer– or both?’ Generally my language is very neutral. And I ask it of everyone. I never assume. (Helen, Halifax)

### Sexual and gender identity do matter

When physicians in the study spoke of LGBTQ social identity (something beyond sexual practices) mattering to patient care, it was almost always in the context of holistic care and recognizing the effects of discrimination.

#### It matters to holistic care

Physicians described the importance of knowing and understanding the whole person in family medicine: “Really trying to understand the patients’ ideas about their health issues, their feelings, their expectations” (Oliver, Vancouver). Harold (Vancouver) suggested that if a patient does not disclose his or her gender or sexual identity to a physician, they lose out in terms of patient care, “because if you don’t understand the context of the dynamics within their environment, that’s a slight loss.” Some physicians insisted a patient’s sexual or gender identity “always matters” (Liza, Halifax), because knowing the patient fully allows the physician to be “more of a complete physician to them” (Joan, Halifax). Others suggested non-disclosure of something so fundamental hints that other significant information may also be hidden, compromising care:

For a long term therapeutic relationship, if they feel they need to hide a significant portion of their life, it’s unlikely that they’re going to feel comfortable with disclosing all the important things and having all of that factoring into, you know, my ability to provide them the best clinical advice. (Karen, Vancouver)

#### It matters because of homophobia, transphobia

Some participants discussed the ways homophobia and transphobia might affect patient’s lives and health, as well as health care. They raised the potential health effects connected to the stresses of ‘coming out,’ potential estrangement from family, and stigma and social exclusion:

They are a stigmatized and discriminated-against group. They just are... There has to be an acceptance of where they are in society. And so, connecting them with peers; connecting them with groups; speaking to them about those gives them a resource and a place where they can have a sense of belonging and support. That’s important. (Helen, Halifax)

Some pointed out concerns specific to health care such as LGBTQ avoidance of health care, denial of same-sex relationships in medical decision-making, and potential intolerance among health professionals. Several participants referred LGBTQ patients only to specialists they believed would not be overtly prejudiced.

### It matters, yet it doesn’t matter..

Some of the most intriguing discussions in our interviews with physicians revolved around ideas of ‘it matters, yet it doesn’t matter.’ They expressed complex balances in their everyday care between recognizing that membership in particular social groups does affect health, and health care, while also recognizing that each health care encounter, and each patient, is unique.

#### An inevitable tension between social group and individual

One participant for example, spoke in detail about how health care for LGBTQ women needs to be different, then went on to describe how they are really the same as everyone else:

For queer women in general, smoking and drinking is a risk factor. Deciding on whether or not they want to have a child is another issue. And then the issues that go into having a child as a two-women couple... How are you going to get pregnant? Are you going to adopt?”... They have the same issues that everybody else has. You know, is the relationship working out? And that is a bit nuanced, because it’s female-on-female, but we all have love affairs and breakups and all those things. So they have the same issues that everybody else has. (Victoria, Vancouver)

Others noted that LGBTQ identity affects interactions in important ways, yet but the facts of many health issues are unaffected. For example:

We are supposed to see things objectively ... ready for the fact that everybody’s different and you might need to change how you interact with them, in order for it to be most successful and most comfortable. But at the same time, to really be looking for, as much as possible, what are the hard and fast, black and white things going on here? You know? Does this person have a heart murmur or not? (Karen, Vancouver)

One physician said that clearly sexual or gender identity matters to some issues in health care, but is not really relevant when, “Somebody has a rash or a sore bone or joint, or pneumonia or a cold or a lot of these other things that are fairly sort of physical and straightforward and isolated in nature” (Mary, Vancouver).

The tension between recognizing the potential effects of social group membership, yet individual differences within groups, and the physical commonalities across all groups makes effective work with diversity challenging. One participant noted that treating all members of a group the same way may be tempting, but is inadequate: “Boiling it down, a lot of people would like to have a handbook on how to deal with queer people, or how to speak to Chinese people. But it’s not that easy” (Karen, Vancouver).

#### Challenging ‘everyone is an individual’

A few participants argued that difference matters yet does not matter, directly challenging the notion that treating every patient as an individual is the optimal way to address diversity. Mary (Vancouver) said she once dismissed the idea of attending to different socio-cultural groups, preferring to “meet every individual person on their own terms.” Eventually she came to believe that without some kind of sensitivity training, “you may not be able to be sensitive to all of an individual’s potential issues.” She highlighted the tension between individual and group differences, noting that emphasis on group membership can lead to stereotyping and inaccurate assumptions, yet sensitivity to group membership can highlight valuable questions to ask an individual:

If you don’t know about some of those potential issues that people may bring in with them, then it’s really hard to actually be sensitive and imaginative enough to ask them everything that you need to ask them... There’s just that tension I guess, between learning about different groups of people, and finding ways to use that as a starting off point for exploration of differences, versus assuming that someone falls into a group. (Mary, Vancouver)

Mary went on to argue for the importance of knowing something about a socio-cultural group, to sensitize the practitioner about possible questions to raise, while not assuming you know everything about each individual member of that group.

## Discussion

Denying the relevance of sexual or gender identity to health care, except perhaps for specific health issues, some physicians strove to avoid labels and to treat everyone as an individual, in order to ensure equity. A counter stance was suggesting that a patient’s LGBTQ identity is important to providing holistic care, and possibly because social marginalisation has health effects. A third stance was to exploit the tension, recognising that LGBTQ identity both matters and does not matter, all the time. This is a complex tension, using recognition of group membership as a means to draw awareness to possible health concerns and appropriate questions for each individual.

When *does* LGBTQ identity matter to health care? Does it only matter for some health issues, but not for others? Does it only matter to actual practices – with whom one has sex? What about a single person who is not currently sexually active? Does the fact that she identifies as lesbian matter? Does sexual or gender identity matter to holistic health care? The tension around this, well-articulated by some participants, is reminiscent of a poem by African American Pat Parker, “For the White person who wants to know how to be my friend.” It opens, “The first thing you do is to forget that I’m black./ Second, you must never forget that I’m black.”^29^

### It’s just a sore throat!

Socio-cultural identities – and the privileges, oppressions, marginalizations, histories, knowledges and areas of ignorance connected to those identities – infuse every aspect of social interaction. When someone sees a physician, their LGBTQ identity should both always matter, and not ever really matter. After all, a sore throat is a sore throat is a sore throat. Yet LGBTQ identity matters because of dominant, normative assumptions. Many of our participants felt very strongly that they did not want to make any assumptions about patients. They described being flustered and embarrassed when they were ‘caught’ mistakenly assuming a patient was heterosexual.

Of course, it is not possible to be assumption-free and neutral.^30^ Humans make assumptions all the time. The position sought after by most physicians – not-labeling, not-judging, not-assuming – in fact means employing ‘unmarked,’ unquestioned, dominant assumptions: heteronormativity and gender normativity. Heteronormativity refers to the assumptions and institutional practices that construct everyone as heterosexual until proven otherwise and that heterosexuality is the normal – indeed only thinkable – sexual orientation. Those who are not heterosexual are cast as deviant, abnormal, lesser. The pervasive assumption of heterosexuality renders other sexual orientations invisible or marginal in health care.^31^ Similarly, gender normativity refers to the pervasive assumptions that there are two distinct genders and everyone fits neatly and uncontestably into one or the other. Normative assumptions about binary gender categories erase not only transgender people, but also those who identify as neither men nor women.

Seeking to treat everyone as an individual, making no assumptions, in fact leaves hetero- and gender normativity unchallenged. The ‘neutral’ patient with just a sore throat – her sexual orientation may not be relevant to diagnosis or treatment, yet it matters because health care providers are almost always assuming (without ever thinking about it) that she is heterosexual and gender normative. While physicians tend to hold themselves individually responsible for making no assumptions (and individually culpable if they ‘slip up’) heteronormativity and gender normativity are part of the very air that we breathe. Such assumptions are inevitable unless consciously countered.

### Individual vs. social differences

In health care both individual and social group differences matter. Yet the focus on individual differences is not balanced by equivalent attention to social differences. Physicians are afraid of stereotyping; yet avoiding that pitfall by refusing to recognise social categories and identities denies an important influence on health and health care.^30^ Ignoring social differences does not erase their patterned, generalizable influence on experiences, life chances, and health (care).

Generalisations are not the same as stereotyping and discrimination. Generalisations allow physicians to take into account the possible effects of shared experiences that arise from marginalisation and discrimination. They bring together group-specific observations and experiences. They suggest difference, not deficit. Stereotypes are an end point for understanding a person, limiting rather than broadening understanding, and applying group tendencies inflexibly to all members of the group. Generalisations are a starting point for understanding an individual, sensitising physicians to possible patterns, and potentially valuable questions. Echoing Pat Parker,^29^ we would say to physicians and medical students, “The first thing you do is to forget that I’m queer. Second, you must never forget that I’m queer.”

The training for LGBTQ care these physicians experienced was minimal at best. There are guidelines for teaching LGBTQ health care in medical schools today.^12^ Yet given the perceptions expressed in our sample, it seems at least as important to start with the notion that LGBTQ identity actually matters. Ideally, education could help medical students to grasp the differences between generalising and stereotyping, enhancing awareness of the patterned ways that heteronormativity and gender normativity shape health and health care, whether they choose to attend to that or not. Eliciting student attitudes toward LGBTQ people in a safe way^32^ is an excellent start. It is equally important to examine where those attitudes and perceptions come from; individual guilt and feeling bad are not helpful for practice. When learners understand that social messages are internalized inadvertently they can begin to learn ways to counter them.

### Limitations

This study is limited by using a self-selected sample, which may mean participants had particular agendas of their own not accounted for in the analyses. It was also a relatively homogenous sample. The study is inherently limited by using self-reported beliefs and practices, which may or may not match people’s actual practices. Future research that uses observational techniques would be valuable, though tricky ethically.

## Conclusion

We found that physicians expressed one of three major approaches to incorporating sexual/gender identity into care for LGBTQ women: (1) makes little or no difference; (2) matters deeply for holistic care; (3) it both matters and doesn’t matter. The common stance that sexual/gender identity matters little if at all means physicians may be ignoring important aspects of social group memberships that affect health and health care. Helping students to understand the importance of generalizations, and the difference from stereotyping, may be an important addition to curricular efforts to improve education concerning LGBTQ health care.
